# Enhancing decellularized vascular scaffolds with PVDF and PCL reinforcement: a fused deposition modeling approach

**DOI:** 10.3389/fcvm.2023.1257812

**Published:** 2023-11-29

**Authors:** Kirill Yu. Klyshnikov, Maria A. Rezvova, Nikita V. Belikov, Tatiana V. Glushkova, Evgeny A. Ovcharenko

**Affiliations:** ^1^ Department of Experimental Medicine, Laboratory of Novel Biomaterials, Research Institute for Complex Issues of Cardiovascular Diseases, Kemerovo, Russia; ^2^ Department of Biomedical Technical Systems, Laboratory of Medical Robotics and Biomaterials, Bauman Moscow State Technical University, Moscow, Russia

**Keywords:** biocompatible polymers, PVDF, PCL, external reinforcement, vascular scaffold, thermal extrusion, decellularized xenogenic scaffolds

## Abstract

**Background:**

Decellularized xenogenic scaffolds represent a promising substrate for tissue-engineered vascular prostheses, particularly those with smaller diameters (<6 mm). Despite their benefits, a notable limitation presents itself during decellularization, namely, the diminished mechanical strength that introduces the risk of aneurysmal dilations in the early post-implantation period. This study introduces a strategy for modification the mechanical properties of these biological scaffolds through the forming of an external polymeric reinforcement via thermal extrusion.

**Methods:**

The study utilized scaffolds fabricated from bovine internal mammary arteries through decellularization and preservation. The scaffolds were divided into subgroups and reinforced with polymeric helices made of Polyvinylidene fluoride (PVDF) and Polycaprolactone (PCL), *n* = 5 for each. An experimental setup for external reinforcement coating was designed. Computed microtomography was employed to obtain accurate 3D models of the scaffolds. Mechanical properties were evaluated through *in vitro* uniaxial tension tests (Z50, Zwick/Roell, Germany), compliance evaluation and numerical simulations (Abaqus/CAE, Dassault Systemes, France) to investigate the effect of external reinforcement on aneurysm growth.

**Results:**

Using a double-layer helix for the reinforcement significantly enhanced the radial tensile strength of the scaffolds, increasing it up to 2.26 times. Yet, the comparison of vessel's compliance between two reinforced and the Control scaffolds within the physiological pressures range did not reveal any significant differences. Numerical simulation of aneurysm growth showed that thin-walled regions of the Control scaffold developed aneurysmal-type protrusions, bulging up to 0.7 mm, with a substantial degradation of mechanical properties. In contrast, both PVDF and PCL reinforced scaffolds did not exhibit significant property degradation, with deformations ranging 0.1–0.13 mm depending on the model, and a maximum decrease in the modulus of elasticity of 23%.

**Conclusion:**

The results of the study demonstrated that the external polymer helical reinforcement of decellularized scaffolds via thermal extrusion enables a controlled modification of mechanical properties, notably enhancing radial strength while maintaining sufficient compliance within the physiological pressure range. A series of *in vitro* tests demonstrated the consistency and potential of this approach for decellularized xenogenic scaffolds, a concept that had not been explored before.

## 1. Introduction

Biologically-derived decellularized vascular scaffolds show promise in addressing the challenge of donor material shortages in vascular interventions (1–3). This approach assumes a minimal immune-inflammatory response to the implant due to the chemical exclusion of antigens while preserving the extracellular matrix of collagen, elastin, and glycosaminoglycans (1). The resultant scaffold is ready for cell colonization as a base for tissue engineering constructs or for use as an autonomous entity with potentially high biocompatibility (3). It is suggested that such vascular scaffolds outperform synthetic vessels in terms of hemocompatibility and provide a more physiological mechanical match between the prosthetic graft and the host artery (4, 5). Additionally, they may provide a valuable alternative to small-diameter vessel (<6 mm) autografts, where polymeric vessels are not applicable due to their high thrombogenicity and compliance mismatch (3, 6, 7). However, the majority of commercially available decellularized scaffolds [such as Artegraft® (8), ProCol® (9), and SynerGraft® technology (10)], are used only in niche applications (1): for hemodialysis access or for bypass procedures on peripheral vessels. Such a limited scope of application for these products is dictated by a specific complication that prevents them from becoming a “gold standard” for small-diameter artery prosthetics or competing with more technologically advanced polymeric ePTFE or Dacron grafts (6). This complication is an aneurysm formation (1), which results from the degradation of the mechanical properties of the vascular wall due to aggressive treatment—decellularization (3, 11, 12). A series of review articles specifically focus on this aspect, primarily based on the results of using the aforementioned commercial products (1, 13). In theory, seeding these scaffolds with recipient cells to produce the extracellular matrix and restore the required mechanical strength of their wall should solve the issue. However, in practice, this process develops slowly, and the vascular prosthesis is exposed to systemic blood pressure immediately after implantation. Consequently, aneurysm formation, especially in the early period, is the key reason for these scaffolds’ failure in clinical practice (14).

A more promising approach to enhancing the mechanical properties of decellularized scaffolds involves creating an additional external layer capable of compensating for these deficiencies, thereby delaying or entirely eliminating the risk of aneurysm formation. One potential solution to this issue is to reinforce the vascular wall with a bioinert polymeric or metallic framework (15, 16). However, such reinforcements could compromise graft compliance, which reflects the vessel's ability to dilate with increasing transmural pressure, a feature critical for maintaining normal vessel hemodynamics (17). Additive manufacturing principles, such as 3D printing, electrospinning, or thread wrapping—which are actively employed in biomedicine (18)—could provide a more adaptive and personalized external reinforcement layer compared to a mesh. These technologies can more accurately reproduce the external relief of biological scaffolds, which exhibit large variability (15). Several experimental studies in the literature have reported the formation of reinforcing spirals for synthetic vascular grafts, applied using a printer (19, 20), suture material threads (21), and attempts to reinforce biological scaffolds with electrospun PCL (22). In the present work, we demonstrate the reinforcement of decellularized biological scaffolds with helices obtained by thermo-extrusion methods, and present the *in vitro* study results of such constructs. This approach enables the replication of the complex external landscape of the scaffold by the helix and permits the use of various polymers for its formation that do not require highly toxic organic solvents used in dissolving certain polymers for electrospinning (23). The primary motivation for such reinforcement is an attempt to increase the radial stiffness of the scaffold to prevent vascular wall bulging in the early post-implantation stage while maintaining longitudinal stiffness. That is why a spiral design was chosen for the reinforcement geometry. This study also presents a series of bench and numerical experiments that explore the change in mechanical properties of such a hybrid structure in comparison to the original non-reinforced scaffolds.

## 2. Materials and methods

### 2.1. Object of the study

Scaffolds for this study were fabricated from bovine internal mammary arteries, featuring an inner diameter of 5 mm, by a process of decellularization and subsequent preservation using diglycidyl ether of ethylene glycol, conducted by “NeoCor” LLC (Russia). This procedure followed a standard protocol (24), in which native vascular samples were incubated in a 1% sodium deoxycholate (DOA) solution (mass/volume) with a 0.1 M Tris-HCl buffer. This incubation period lasted 16 h at a constant temperature of 37°C with continuous stirring. Following this, the samples underwent a thorough rinse with the 0.1 M Tris-HCl buffer (pH 8.3) for a duration of 24 h, after which the scaffolds were preserved with diglycidyl ether of ethylene glycol. Based on the decellularization protocol we chose and the length of the vascular segments being about 7.5 cm, we did not use the perfusion method in this study.

Native and decellularized vascular samples were frozen and serial cryosections were prepared using a cryotome Microm HM 525 (Thermo Scientific, Waltham, MA, USA) for hematoxylin and eosin (H&E) staining. Also, cryosections were fixed in a 4% paraformaldehyde solution for 10 min. To block non-specific binding, samples were incubated with a 1% solution of bovine serum albumin for 1 h. Then, they were treated with antibodies against type IV collagen (ab6586, Abcam, Cambridge, UK) or antibodies against elastin (E4013, Sigma-Aldrich, St. Louis, MO, USA). Specimens were incubated with the primary and secondary antibodies at room temperature for 1 h each. At all staining stages, samples were intermittently washed with phosphate-buffered saline containing 0.1% Tween. Nuclei were counterstained with DAPI (10 μg/ml, D9542, Sigma-Aldrich, St. Louis, MO, USA) for 30 min. Stained specimens were mounted using ProLong mounting medium. Specimens were examined using a confocal microscope LSM700 (Carl Zeiss, Oberkochen, Germany). To estimate the residual DNA content native and decellularized vascular samples were frozen and lyophilized before being incubated in a papain digestion solution containing 2.5 U of papain, 5 mM cysteine HCL, and 5 mM Ethylenediaminetetraacetic acid (ETDA) in water at 60°C for 24 h (all reagents from Sigma-Aldrich, USA). Quantitative measurements of the total DNA content were then determined by the Quant-IT PicoGreen dsDNA Assay Kit (Invitrogen, San Diego, CA, USA) (*n* = 5 for each group), following the manufacturer's specifications.

**Figure 1:**
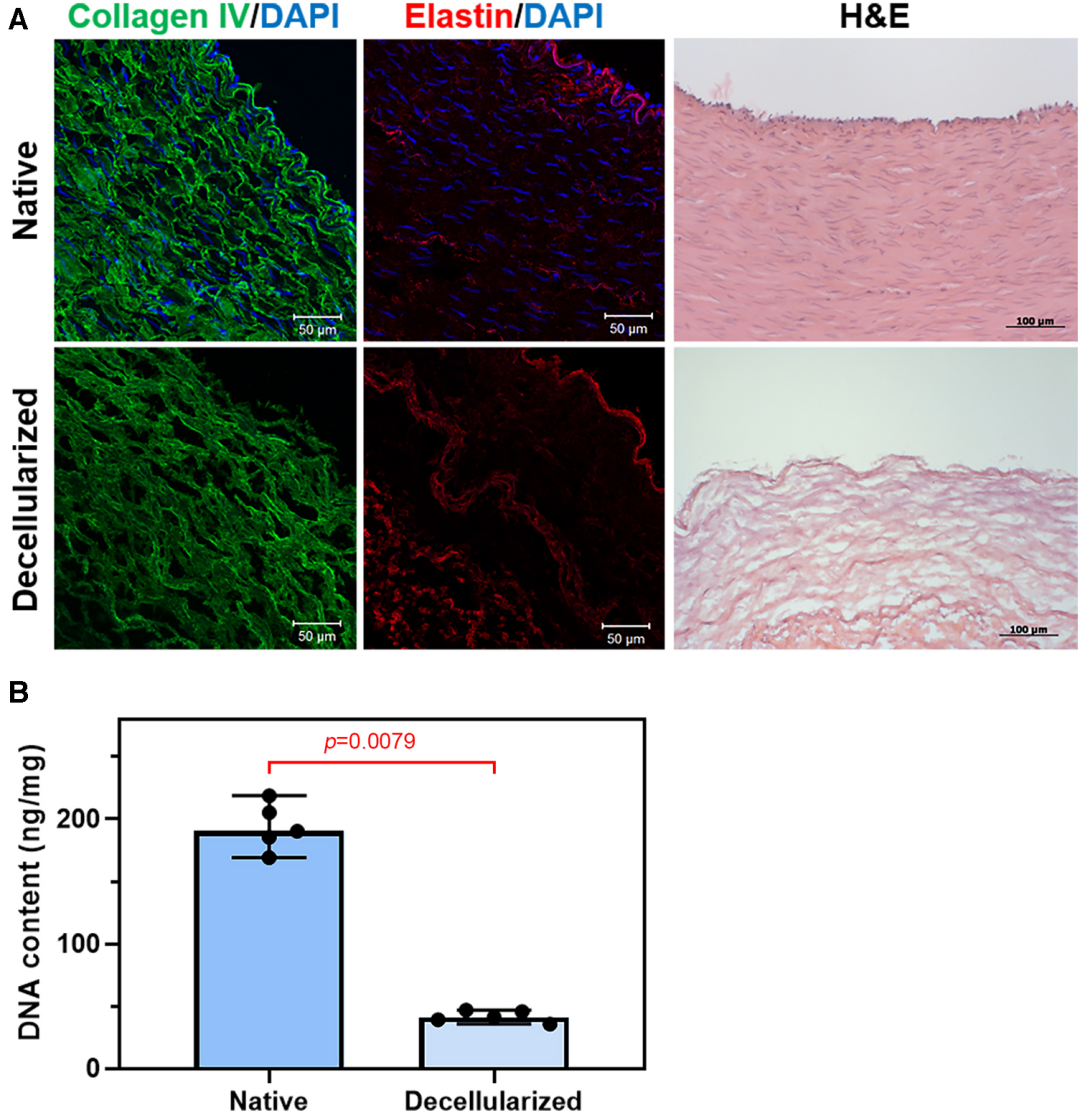
Decellularization of bovine arteries: (**A**) representative immunofluorescent staining and H&E staining of the native and decellularized bovine artery cross sections, collagen IV (green), elastin (red), and the nuclei of all cells were stained with DAPI (blue); (**B**) DNA content of bovine artery before and after decellularization.

Bovine artery was successfully decellularized using the decellularization technique. H&E and DAPI staining showed that the nuclear content of the tissue has been successfully removed (Figure 1A). At the same time, the structure of collagen and elastin fibers of vascular prostheses was not significantly changed. Relative DNA content decreased from 190.2 [177.3–212.0] ng/mg for native artery to 41.2 [37.3–46.1] ng/mg for decellularized sample as shown in Figure 1 (Figure 1B).

Utilizing the process, 15 scaffolds were obtained and divided randomly into three subgroups: Control samples (*n* = 5), samples for reinforcement with polymeric helix №1 (*n* = 5), and №2 (*n* = 5).

Two biocompatible thermoplastic polymers were selected as reinforcement materials:

№1. Polyvinylidene fluoride (PVDF, Sigma Aldrich, USA, Mw = 530,000 Da), a non-degradable reinforcement intended for permanent and prolonged aneurysm protection.
№2. Polycaprolactone (PCL, Sigma Aldrich, USA, Mw = 80,000 Da), used as a temporary biodegradable reinforcement during the initial stages of scaffold functionality when colonizing cells are unable to provide sufficient mechanical strength and structural integrity to the vascular construct. PCL is extensively utilized in tissue engineering due to its biodegradable and biocompatible nature (25).

The granulated polymers were extruded into filaments of 1.75 mm in diameter, suitable for application in the experimental setup based on the fused deposition modeling (FDM) method.

### 2.2. Experimental reinforcing setup

**Figure 2:**
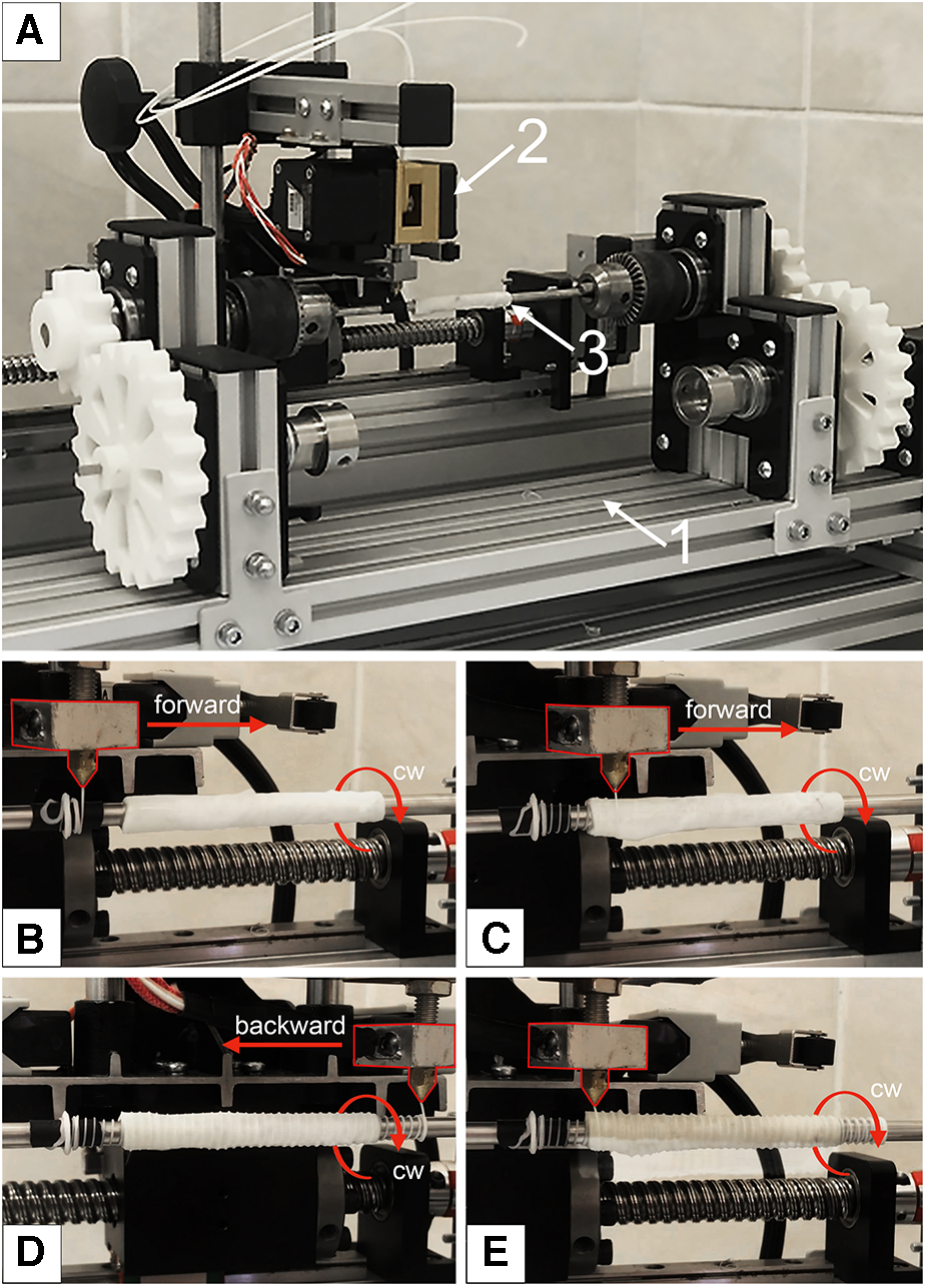
Experimental equipment for a reinforcing layer forming: (**A**) main view, where 1—the body; 2—the mobile carriage with an extruder; 3—the winding shaft with mounted decellularized xenogenic scaffold; (**B**) initial position of the extruder for forward movement; (**C**) the beginning of the extruder's movement and the formation of the first helix; (**D**)—the extruder's end position after applying the first reinforcing spiral; (**E**) completion of the formation of the second layer of reinforcing polymer thread.

**Figure 3:**
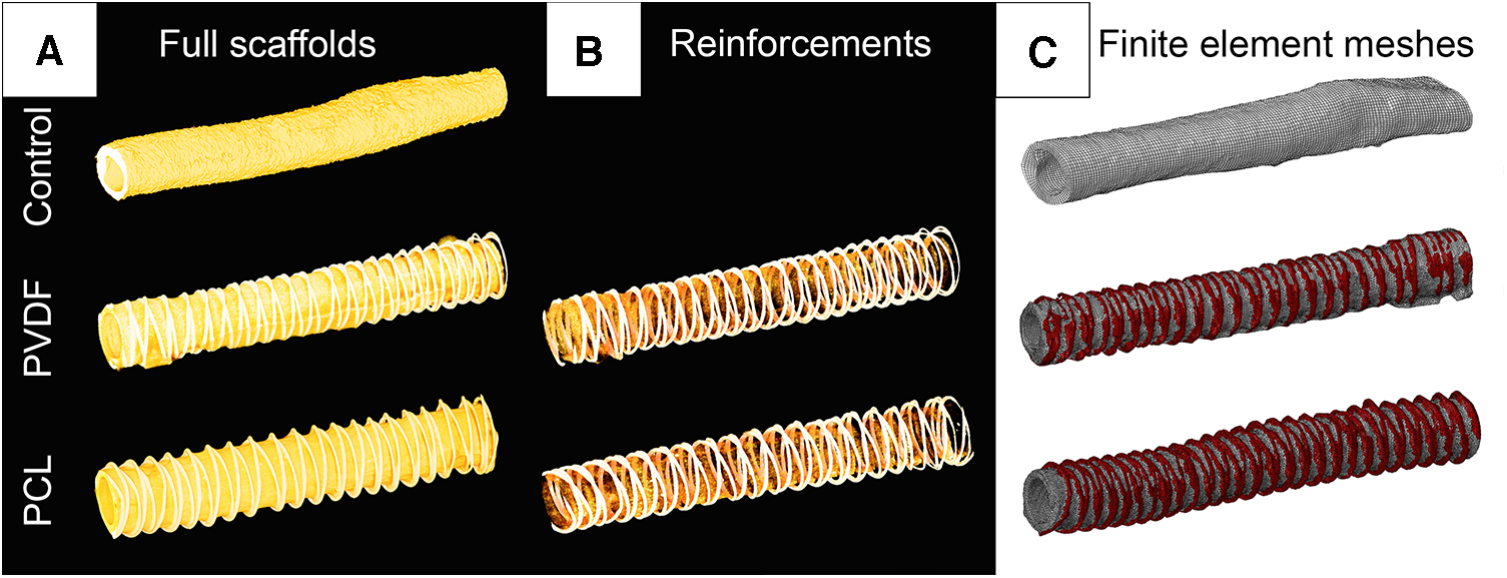
Visualization of computer microtomography of scanned vascular scaffolds and the finite element mesh reconstructed based on them: (**A**) scaffolds in the full spectrum of radiological density; (**B**) visualization of only the polymer spiral; (**C**) visualization of the finite element mesh in the abaqus/CAE environment with highlighted material for the biological base and polymer thread.

Experimental equipment was designed for the external reinforcement coating, which enables controlled application of the 3D helix (Figure 2). The basic components of the setup are:

1. The body (Figure 2A-1), consisting of a table with adjustable legs on which the carriage, winding shaft, and electronics are mounted. The positions of the carriage and the shaft are adjustable relative to each other.
2. The mobile carriage with an extruder (Figure 2A-2). The carriage is driven by a ball-screw drive mechanism and a stepper motor. Controlled by custom software, the extruder heats up and forming a polymeric reinforcing helix via the FDM principle.
3. The winding shaft (Figure 2A-3) is located underneath the mobile carriage. The scaffold to be reinforced is mounted on it. The winding shaft is a rod that matches the inner lumen of the vessel (5 mm) and measures 200 mm in length. The winding shaft rotates clockwise, synchronously with the movement of the carriage, driven by a stepper motor and gear system.

### 2.3. Reinforcing

The decellularized scaffolds (*n* = 15) were divided into segments of 15 mm and 60 mm and split into three subgroups: non-reinforced Control; samples for the PVDF helix; and samples for the PCL helix. Thus, each subgroup was represented by five 15 mm samples and five 60 mm samples. Scaffolds were reinforced with an appropriate polymeric helix using the experimental FDM setup (26). The thickness of the final applied helices was measured using a thickness gauge Mitutoyo 547 (Mitutoyo, Japan) with a resolution of 0.01 mm at 10 different points for 15 mm samples (*n* = 5 for each group). The regularity of the spiral application was visually examined using an Olympus SZ51 binocular (Olympus, Japan) with a 2× magnification and an integrated measurement scale in the lens. For this, we measured the distance between identical parts of the spirals (typically peaks) 10 times for each sample. Additionally, during this examination, we visually assessed the quality of the spirals—checking for cracks, defects, and breakages.

### 2.4. Computed microtomography

For subsequent numerical experiments, accurate three-dimensional models of the investigated scaffolds were obtained. A single random 60 mm sample from each subgroup was scanned using the “Orel-MT” microtomography equipment (Russia). Scanning characteristics included: voltage 80 kV; current 48 μA; frame exposure time 0.667 s; number of frames in projection 3; number of projections 1,200; angular step 0.3 degrees; voxel size 25.4 μm. The obtained tomographic slices were imported into the Mimics software (Materialize, Belgium) for reconstructing the volumetric 3D-facets (Figure 3C) of three vascular scaffolds: Control, PVDF, and PCL. All models were STL grids of tetrahedral elements (*n* = 538–808 thousand), suitable for the finite element analysis of aneurysm formation.

### 2.5. Compliance

**Figure 4:**
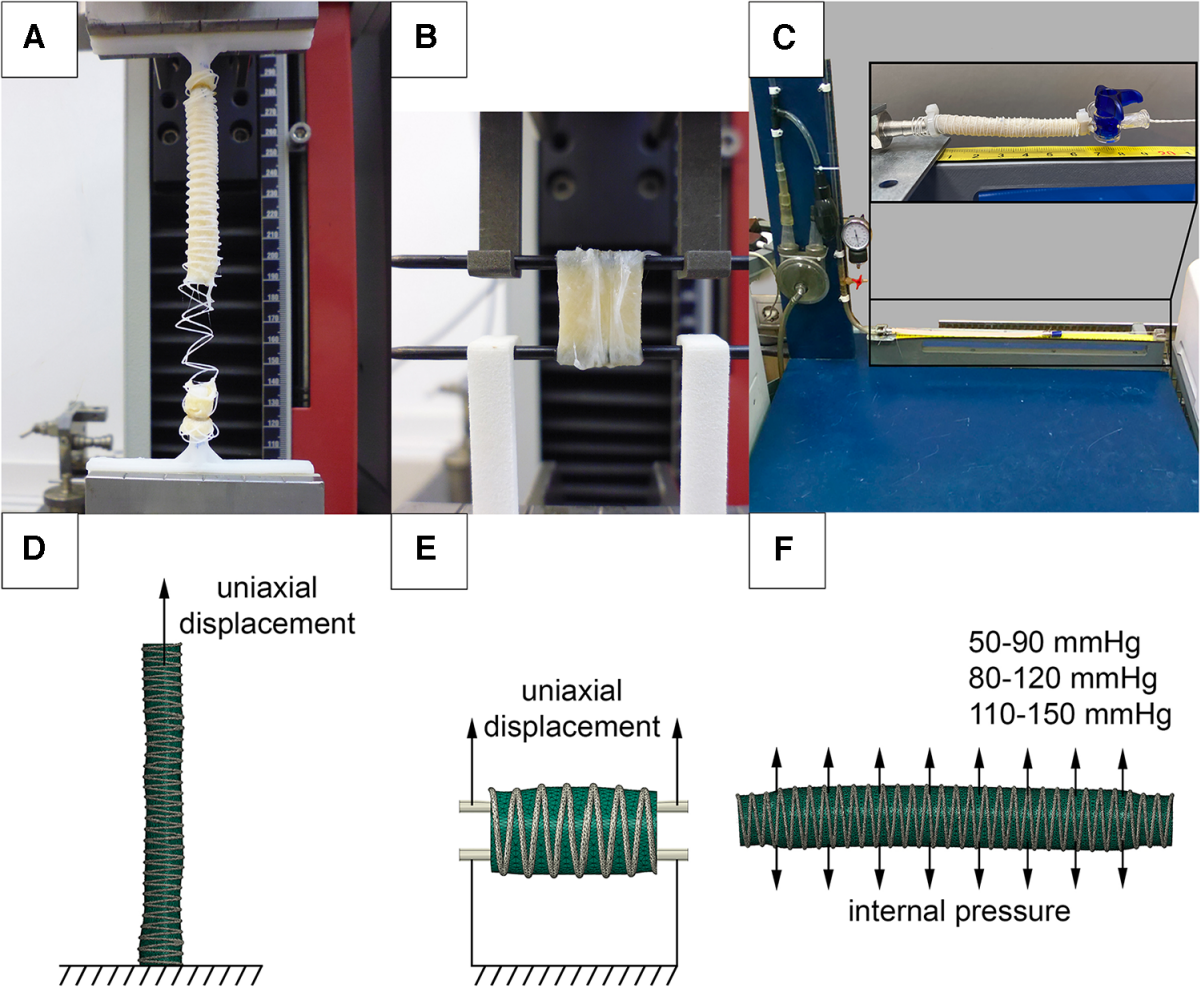
Investigation of the mechanical properties of reinforced scaffolds: (**A**) uniaxial elongation of vascular prostheses in the longitudinal direction; (**B**) in the transverse direction; (**C**) setup for assessing compliance when applying internal pressure; (**D**) numerical experimental model for verifying elongation in the longitudinal direction; (**E**) in the transverse direction; (**F**) numerical setup for verifying the compliance of vascular prostheses.

The effect of changes in vascular prosthesis compliance was studied *in vitro* on an experimental setup (Figure 4C). For this purpose, 60 mm scaffold samples for the Control, PVDF, and PCL subgroups (*n* = 5 for each) were fixed in mounting sockets. Increasing internal pressure from saline solution was consistently applied in three ranges: 50–90, 80–120, and 110–150 mmHg, corresponding to hypo-, normo-, and hypertensive values. We applied the load statically, starting with the pressure of the lower boundary, then raising it to the upper boundary and bringing it back down. Changes in the external diameter were evaluated using a video camera, with the calculation of the difference between “diastolic” and “systolic” diameters, expressed as a percentage of the minimum.

In parallel, a similar in silico analysis was conducted in Abaqus/CAE (Dassault Systemes, France) to evaluate the influence of the helix on scaffold compliance (Figure 4F). The objects of study were microtomographic 3D-facets. The ends of three-dimensional models with a length of 5 mm were fully restrained from displacement (defined as “encastre” boundary condition), and pressure equivalent to 0.007–0.012 MPa (50–90 mmHg), 0.011–0.016 MPa (80–120 mmHG), and 0.015–0.020 MPa (110–150 mmHg) was applied to the inner surfaces. A polynomial model, obtained from the results of mechanical tests (see below), was chosen to describe the biomechanics of the vascular wall. The solver selected was Abaqus/Standard, and the duration of the load was set to 1 s.

### 2.6. Mechanical properties

To evaluate changes in mechanical properties after reinforcement, an *in vitro* uniaxial tension test was performed. The evaluation of the mechanical properties was carried out using a universal testing machine Z50 (Zwick/Roell, Germany). 60 mm samples (*n* = 5 for each subgroup) were fixed into custom holders and stretched uniaxially at a rate of 20 mm/min (Figure 4A). 15 mm samples (*n* = 5 for each subgroup) were attached to custom clamps and stretched transversely (Figure 4B), similar to the method described by Mi, H.-Y. et al. (27). During the testing, stress-strain curves were recorded. The stop criterion was a 30% drop in the force, registered by the sensor, which was associated with either the rupture of the polymeric helix or the scaffold.

Parallel numerical simulations of scaffold longitudinal and transverse tension were performed using Abaqus/CAE, utilizing the finite element method. The microtomographic three-dimensional models of the decellularized scaffolds were used (one model each subgroup, Figures 4D,E). The goal of such simulation was to validate material models that describe the behavior of the helix and the biological component. For the case of longitudinal stretching, the lower end of the 60 mm scaffold model was fully fixed against movement (“encastre”), and uniaxial displacement (U) was applied to the nodes at the upper end by 60 mm along the Z axis. When simulating transverse stretching, 15 mm models cropped from the long 3D-facets were stretched using two cylinders simulating custom clamps, similar to the *in vitro* mechanical tests. The lower cylinder was fully fixed against movement (“encastre”), and the upper cylinder was moved 20 mm along the Z axis. The interaction between the scaffold and the cylinders was described as surface-to-surface contact. The interaction properties were defined as follows: normal behavior was modeled using ‘hard’ contact, and the tangential direction was represented using a penalty type with a friction coefficient of 0.2. The solver chosen was Abaqus/Standard. During the modeling process, the “stress-strain” results of the natural and numerical experiments were compared for both cases—longitudinal and transverse loading.

### 2.7. Numerical aneurysm growth

Given that actual aneurysm growth *in vivo* is an extremely long and uncontrolled process, a numerical simulation was conducted to verify the idea of external reinforcement and modifying the mechanical properties of the vascular wall to protect against its pathological dilatation. The simulation was conducted in Abaqus/CAE, mimicking the multiple cyclic pressure action. For that purpose, the models for compliance in silico simulations were modified. The ends of the three vascular prostheses (Control, PVDF, PCL), each 60 mm long, were completely fixed against displacement, and pressure, characteristic of peripheral arteries in humans, was applied to the inner surfaces of the models (28). However, in this case, the pressure changed cyclically over the course of 150 virtual cycles. Also, to replicate the process of aneurysm formation, a degradation function of the modulus of elasticity, dependent on the level of plastic deformation, was incorporated into the linear model of the scaffold material (biological part). The yield limit (0.6 mm/mm), at which the stress-strain curve of the material loses its linearity, became the threshold for such degradation activation. This function was realized by integrating the USDFLD subroutine of Abaqus/CAE. It should be noted that to simplify high-cost simulation, we used linear material models, characterized by the modulus of elasticity and the plastic deformation curve, corresponding to the results of natural investigations of mechanical properties. The modulus of elasticity for the biological part amounted to 1 MPa, and the yield limit was 0.6 mm/mm in terms of deformation. Such an approach to the combination of two processes occurring in the material of the vascular wall is a simplified version of the complex biomechanics of aneurysm growth described in the literature (29). Lee EH and Baek S (29) describe two factors—the accumulation of plastic deformation and the degradation of vascular wall properties (primarily, elastin) as mechanisms of pathological vessel dilation. Undoubtedly, the current formulation implements this concept only ideologically—at the level of changing the modulus of elasticity, but for demonstrating the differences between the reinforced and intact vascular scaffold, we consider such simplification not critical.

**Figure 5:**
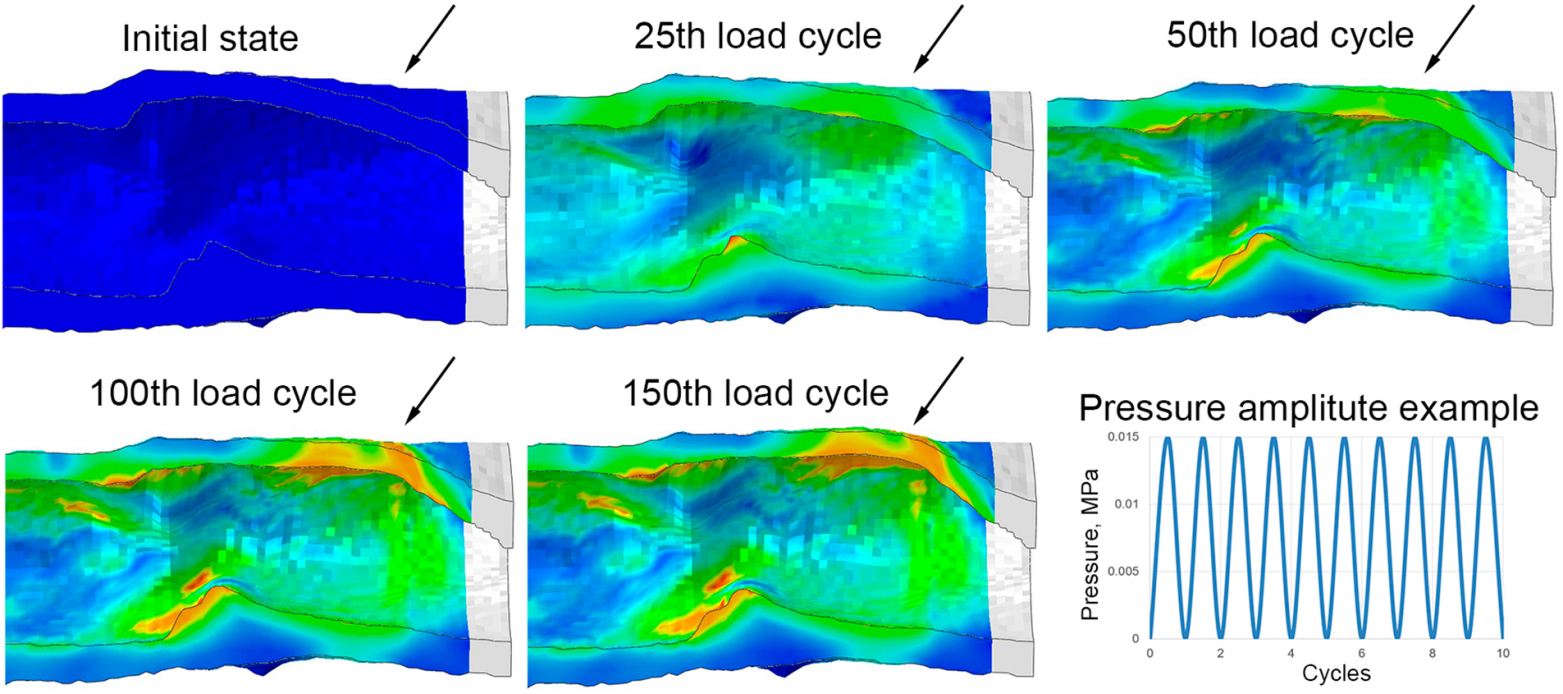
Implementation of the aneurysm formation mechanism in a numerical experiment: an example of the evolution of the wall bulging at one end of the control (without reinforcing) vascular scaffold with the increase in the number of loading cycles. Images are shown in the longitudinal section after the removal of pressure from each cycle (relaxed state). Arrows highlight the parts of the vascular wall that remain bulging after the pressure is released.

Hence, the numerical experiment involved two primary attributes of vascular prosthesis biomechanics: as soon as stress exceeded the threshold of plastic yield, irreversible deformation emerged and simultaneously, the elasticity modulus of the scaffold decreased. In areas where the threshold stress was not surpassed, the effects did not manifest—all deformation was reversible, with no decrease in the wall properties. As a result of such behavior, the elasticity modulus in the model ranged from 100 to 10% relative to the original, individually for each region of the scaffold (more precisely, for exact finite element). In the case of “defective” areas, every subsequent pressure cycle induced a greater accumulation of plastic deformation and a further degradation of the elasticity modulus, leading to a gradual dilatation of the scaffold's vascular wall (Figure 5).

Material models for used polymeric helix were also linear: the elasticity modulus for the PVDF polymer was 458 MPa, and 321 MPa—for PCL, without plastic deformation or degradation of elastic properties implementation. During the simulation the diameter, radial displacement (U1), the amplitude of plastic deformation (PE), as well as the degree of degradation of the vascular wall's elasticity modulus in the most altered region of the scaffolds were measured.

### 2.8. Statistical analysis

GraphPad Prism 9.4.1 software (San Diego, CA, USA) was used for statistical analyses. All continuous variables were presented as the median (Me) with the interquartile range [IQR]. Pairwise comparisons between groups were conducted using the Mann–Whitney *U*-test. The comparison groups for assessing the quality of polymer reinforcement application were samples with PVDF and PCL. For compliance and mechanical properties evaluations, each comparison group was tested against the Control—non-reinforced scaffold samples. A *p*-value < 0.05 was considered statistically significant. All reported *p*-values are two-tailed.

## 3. Results

### 3.1. Reinforcing

**Figure 6:**
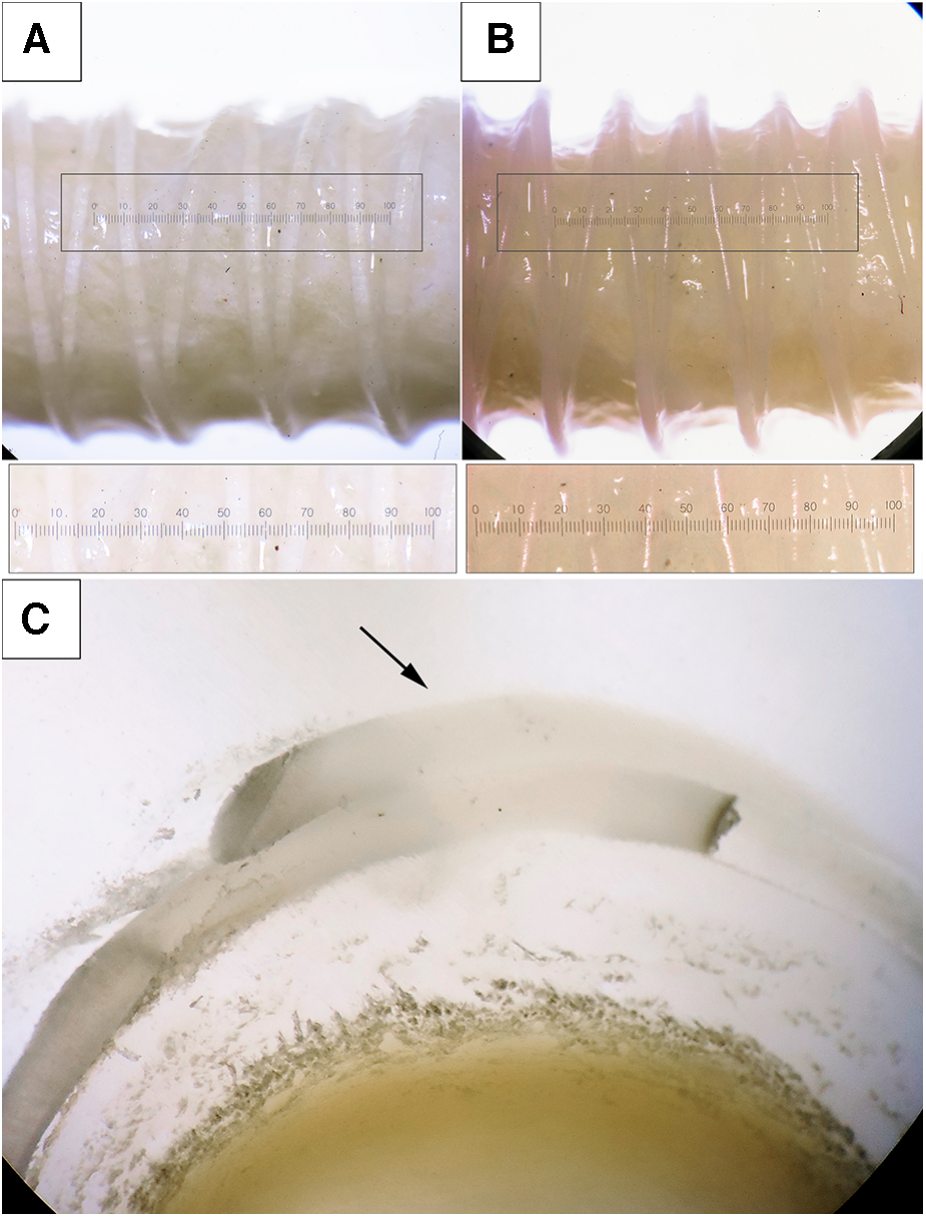
Photographs of the scaffolds with the reinforcing helices, binocular microscope with magnification ×2. (**A**) the reinforcing thread is made from PVDF; (**B**) PCL; (**C**) an enlarged cross-section of a reinforced PVDF vessel, visualization of the fusion of the spirals (magnification ×2.5).

We demonstrated that both polymeric materials (PVDF and PCL) can form a double helical structure that repeats the external contour of the decellularized scaffold. The helical turns were applied uniformly and were fused at points of polymer overlapping (Figure 6C, arrow). However, the fusion of the polymer layer with the biological component did not occur—the helix interacts with the scaffold, embracing it, but does not form a unified structure with it. A visual analysis using a binocular microscope revealed variability in the distances between turns, which equaled 2.25 [1.91–2.51] for PVDF and 2.15 [1.97–2.34] for the PCL variant (*n* = 5 for each group, *p* = 0.94). This variability could be attributed to the varying external surfaces of the biological scaffolds. The thickness of the helical threads was 0.33 [0.28–0.35] mm for PVDF and 0.3 [0.28–0.35] mm for PCL (*n* = 5 for each group, *p* = 0.94). Thus, it can be assumed that the geometric characteristics of the reinforcing helices do not depend on the material—PVDF or PCL. The intragroup variability (the “imperfection” of the helix) is caused by the inaccuracies of the thermal extrusion method and the uneven surface of the scaffold, which the helix is forced to replicate. The measurements confirmed the continuity of the helices without any fractures and critical angles for the polymer threads (Figure 6).

### 3.2. Compliance

**Figure 7:**
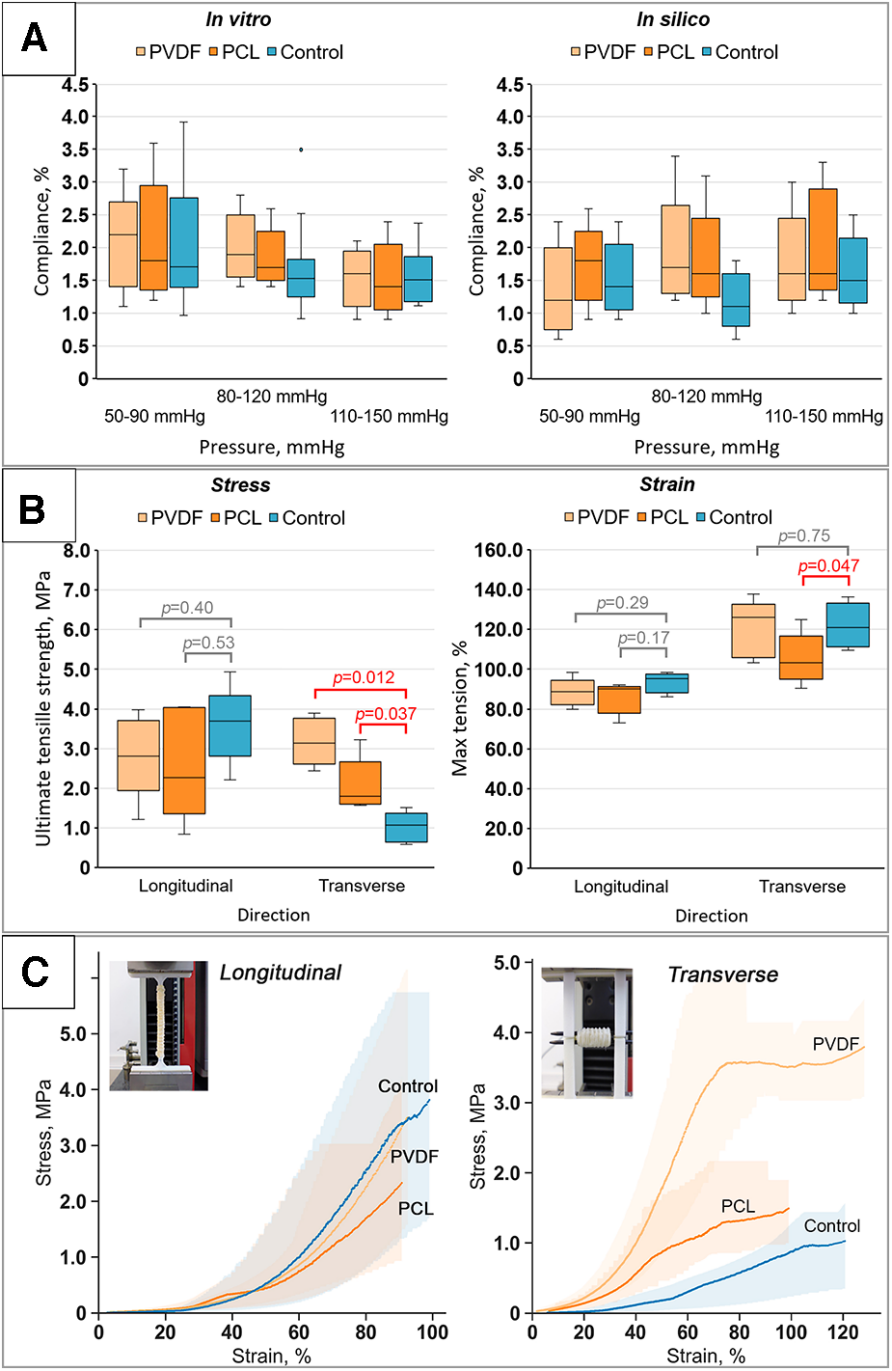
Results of bench tests of the obtained scaffolds: (**A**) results of compliance research *in vitro* and in silico (numerical modeling); (**B**) investigation of mechanical properties for both directions (longitudinal and transverse): tensile strength limit and strain limit; (**C**) stress-strain curves for the samples tested in both directions (longitudinal and transverse) with visualization of data variability (*n* = 5 for each group).

The evaluation of compliance revealed that reinforcement had a minimal impact on the percentage change in diameter within the examined ranges. We did not observe any statistically significant differences in the compliance of reinforced scaffolds compared to the Control for all pressure ranges (the corresponding *p*-value figures are presented in Figure 7A and in Supplementary Material S1). Presumably, this is due to the high variability of results, especially for the Control. Moreover, the loads are relatively low to demonstrate differences between the groups, as only the biological component of such composite constructions is engaged in the mechanical response. However, with a significant increase in loads (see the “Mechanical properties” section below), during which the material tears, the “activation” of the helix occurs and the differences become significant. Similar outcomes were yielded during the computational verification of compliance assessment. However, a comparison of the *in vivo* and computational experiments displayed a decrease in the compliance measure in the latter scenario, particularly within the low-pressure range (50–90 mmHg).

### 3.3. Mechanical properties

The investigation into the mechanical properties of the biological component (Control group) demonstrated high variability in subgroups with a high interquartile range (Figure 7B). The classic nonlinear J-shaped curve of the biomaterial's mechanical characteristics was obtained (Figure 7C) (30), a result of the vascular wall's heterogeneous structure, composed of collagen and elastin fibers.

The evaluation of the mechanical behavior of the reinforced scaffolds revealed the following tendencies:

Upon longitudinal stretching, it was shown that the maximum tensile stress for the Control samples was 3.69 [3.41–3.73] MPa; for samples reinforced with PVDF, it was 2.81 [2.66–3.45] MPa; and for PCL, it was 2.27 [1.86–4.01] MPa. Despite some differences, no statistically significant differences were found between the Control (non-reinforced) and the reinforced vascular prostheses for both PVDF (*p* = 0.40) and PCL (*p* = 0.53) (Figure 7B). In this case, the samples stretched to 95.2 [90–96.6], 90.2 [83–90.6], and 88.6 [84.2–90.4]%, respectively, upon reaching failure. However, these differences also lacked statistical significance. That is, the mechanical properties during longitudinal stretching were not altered by the reinforcing helix.

Fundamentally different results were observed for transverse loading. The stress statistically differed for vessels reinforced with PVDF and PCL compared to the Control group (Figure 7B). As expected, both groups of reinforced scaffolds showed greater maximum tensile strength due to the presence of the polymeric helix. The tensile stress for the Control was 1.07 [0.7–1.23] MPa, while for PVDF and PCL reinforced scaffolds it was 3.14 [2.8–3.64] and 1.8 [1.64–2.12] MPa, respectively (*p* = 0.012 and 0.037 respectively). Mixed results were observed for the strain—for scaffolds with PVDF reinforcement, there was no significant change compared to the Control 121.02 [112.74–129.94] vs. 126.11 [108.28–127.39]% (*p* = 0.17), while samples with PCL, on the contrary, showed a loss of strength, stretching to 103.18 [99.36–108.28]% of their original size (*p* = 0.047). Hence, in this case, there was a modification of mechanical properties.

In addition, we obtained the mechanical properties of isolated polymeric helices for the computational aneurysm growth modeling. It was demonstrated that the median modulus of elasticity of the extruded PVDF polymer was 458 [418–473] MPa, and for PCL—321 [318–344] MPa. For the biological component, an initial section of the curve was isolated—within the range of minor, near-physiological loads, up to 1% deformation—and the modulus of elasticity was calculated for use within a linear model with property degradation. The initial modulus of elasticity was set at 1 MPa.

### 3.4. Numerical aneurysm growth

**Figure 8:**
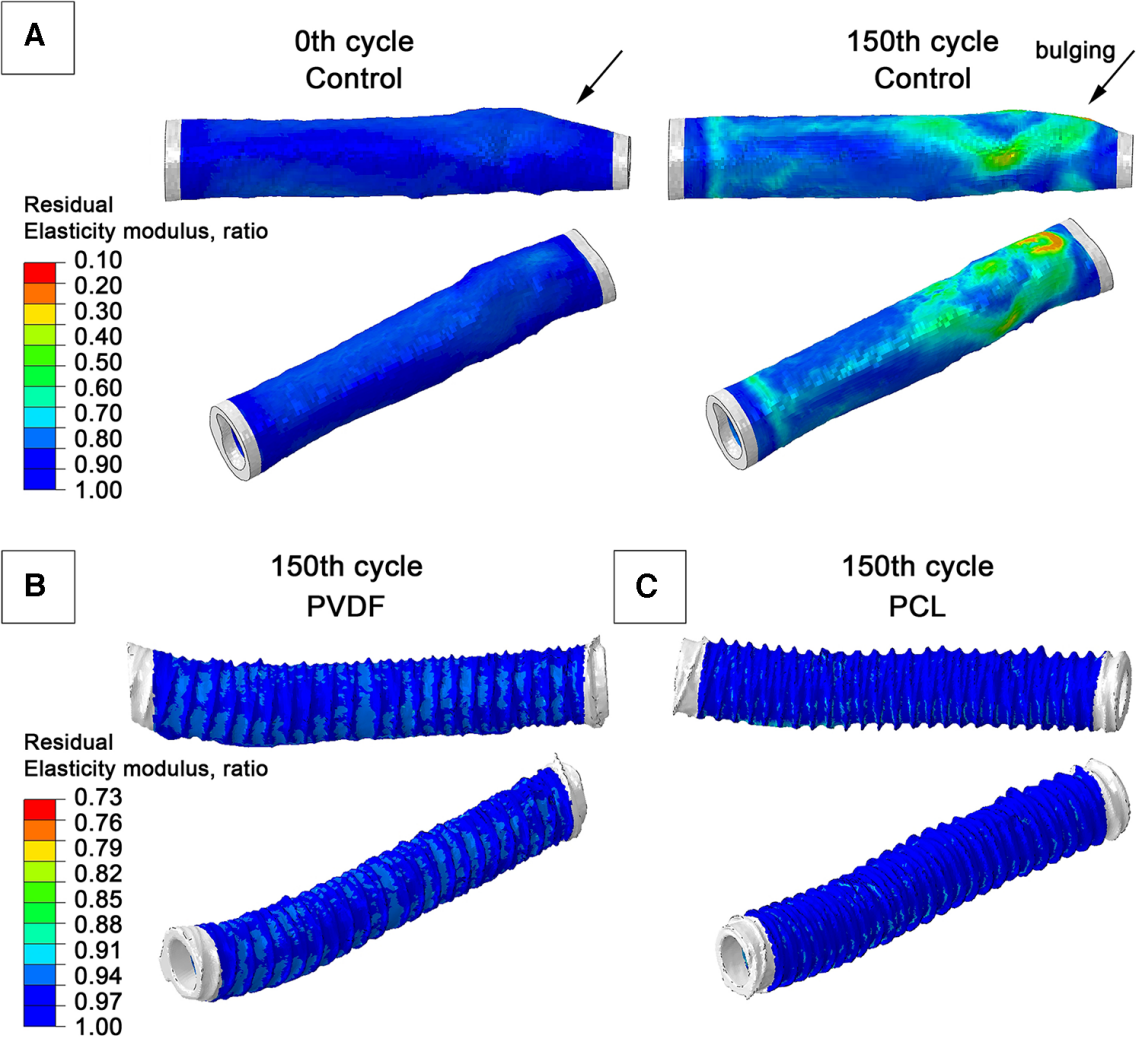
Graphs from numerical modeling, visualizing the degree of degradation of the elastic modulus of the biological component of scaffolds as a result of the impact of cyclical load (a hypothetical 150 pressure application cycles): (**A**) model of a vascular prosthesis without reinforcement; (**B**) model reinforced with a double PVDF helix; (**C**) model reinforced with a double PCL helix.

Computational modeling demonstrated different behavior between the Control and the reinforced scaffolds in response to cyclic load. The model without reinforcement exhibited an expected gradual accumulation of plastic deformation and a decrease in the modulus of elasticity as the number of load cycles increased. Such changes in the model occurred heterogeneously—depending on the geometric characteristics (primarily thickness) of the scaffold wall. The greatest property changes were observed for areas of minor thickness—by the 150th load cycle, the residual modulus of elasticity decreased to 10% of the initial (i.e., 0.1 MPa with an initial 1 MPa). This resulted in a bulging of the vascular wall in this area, resembling an aneurysm (Figure 8A)—in the radial direction up to 0.7 mm. Such changes were not as dramatic for other areas: after load removal, the residual radial displacement was up to 0.43 mm depending on elasticity modulus degradation. The plastic deformation (max. principal PE value) was at a maximum of 0.55%.

For both reinforced scaffolds, such phenomena were also observed: the modulus of elasticity of the biological part also decreased, but not so manifestly—up to 77% for PVDF reinforcement, and to 87% of the original value for PCL (i.e., to 0.77 MPa and 0.88 MPa, respectively). The maximum residual radial displacement was 0.13 mm for PVDF reinforcement and 0.1 mm for PCL reinforced samples. No accumulation of plastic deformation (max. principal PE indicator) was observed—only 0.15% for PVDF and 0.32% for PCL. The strain plots clearly illustrate the behavioral differences between scaffolds: in the reinforced models, the main load is transferred to the polymer spiral, which helps prevent degradation of the biological component's mechanical properties.

## 4. Discussion

The *in vitro* compliance study revealed two particularities of vascular prostheses’ response to applied internal pressure: (1) the reinforced and Control samples did not differ in the deformation indicator in each pressure group (hypo-, normo-, and hypertensive), and (2) they exhibited a tendency towards decreased compliance of the vascular prosthesis with increasing pressure—transitioning from the hypotonic to the hypertonic values. The first effect could be explained by the substantial contribution of the biological component to this characteristic. The ranges of test pressures used in the study (50–150 mm Hg) are comparatively small to the loads that require the involvement of the reinforcing in the material's mechanical response. Differences in the behavior of the experimental and Control prostheses emerge at larger deformations, whereas at physiological pressure ranges, strain amount to up to 2%. Consequently, the helices “do not engage” in the vascular prosthesis’ response under such loads (up to 150 mmHg). Given that the biological component for all subgroups is identical—the internal mammary artery of cattle, selected and processed using identical technology, the differences are minimal. The second effect—the decrease in compliance when transitioning from the hypo- to the hypertonic range could also be related to the features of the biological component. The connective tissue fibers have a limited “reserve” of stretch, which is “consumed” with increasing pressure. At low pressures, the curved collagen fibers slightly straighten, and at high pressures, considering the notable (110 mm Hg) prestress, the fibers have a limited ability to straighten, which reduces the overall compliance of the vessel wall (31).

The mechanical properties of both polymeric and biological components exhibit two tendencies: (1) low variability in polymer subgroups and, conversely, high variability in the biological subgroup, and (2) statistically significant differences for the transverse direction when comparing reinforced scaffolds pairwise with the Control. The substantial variability in the mechanical characteristics of Control samples is due to their nature—biological tissue is expectedly more heterogeneous in geometric and material properties (32), which ultimately manifest in differences in biomechanics. Polymeric materials, by contrast, exhibit greater structural homogeneity and reproducibility during manufacturing, especially when formed via FDM extrusion. Therefore, the integration of a helical reinforcing component into the vascular prosthesis composition neutralizes the variability of the biomaterial, which we demonstrate by the results of mechanical tests of the assembled prostheses—the interquartile range of all reinforced subgroups (PVDF and PCL) is lower than the interquartile range for the Control.

Another observed effect was the presence of statistically significant differences only for transverse stretching, attributable to the geometric characteristics of the reinforcement (the double helix). This structure, on one hand, does not limit the longitudinal mobility of the scaffold due to the spiral's compression-extension, and on the other, it restricts stretching in the transverse direction due to the fusion of its individual rings. Consequently, in the longitudinal direction, the principal contribution to the mechanical response is made by the biological component—similar for all groups, while for the transverse direction, the main contribution is made by the polymer component—significantly differing in properties between groups (Figure 7B).

Overall, due to the reinforcement, we observe an artificial increase in the anisotropy of the two-component vascular prosthesis after the polymer layer forming. For instance, reinforcement using PCL increased the radial stiffness of the entire vessel by 1.39 times, and PVDF by 2.26 times, without significant changes in longitudinal stiffness. This conclusion aligns with the findings of similar studies: the formation of a bi-layered structure can enhance the mechanical properties of decellularized biological scaffolds. Gong W. et al. distinctly demonstrate that external reinforcement can significantly increase radial stiffness (up to 1.8 times) relative to the decellularized scaffold, and also relative to the native vessel (up to 1.25 times) (22). Notably, in this study, the authors demonstrate an increase in longitudinal stiffness as well—up to 2.6 times relative to decellularized samples, which is due to the use of electrospinning technology for reinforcement with full involvement of such a “shell” in the mechanical response. Similarly, Janke H.P. et al. (33) demonstrate a significant change in the mechanical properties of a collagen matrix reinforced with a double polymer spiral compared to an unreinforced one (33). The modulus of elasticity increased significantly. Meanwhile, full integration of the spiral into the vascular prosthesis changed properties in both longitudinal and transverse directions.

Our numerical assessment of the aneurysmal changes in the scaffold wall supports the concept of reinforcement, which is prone to property degradation under prolonged alternating loads. With an equal duration of load (a hypothetical 150 cycles), both reinforced scaffolds demonstrated moderate degradation of the biological component's properties. Meanwhile, some areas of the Control model underwent significant changes in material properties (elastic modulus) and its geometry, with aneurysm-like bulging. Undoubtedly, based solely on numerical modeling, it cannot be claimed that reinforcing the scaffold with a spiral will have a definitive protective effect. More detailed *in vitro* tests and validation of numerical modeling should be the next step in researching this reinforcement concept. Nevertheless, we believe that such an approach has the potential to “protect” against aneurysm formation.

In general, the approach of vascular wall mechanical properties degradation with the accumulation of plastic deformation is actively considered by researchers as a model for aneurysm growth (34). The basic idea of this approach involves the destruction of extracellular matrix components and the subsequent change in the properties of the scaffold's wall as a result of hemodynamic factors’ influence [high or low wall shear stress (35)] or inflammation and proteolytic enzymes (MMP-2 and MMP-9) action (36). It should be noted that the degradation model chosen in this study does not account for such deep processes and is much more simpler than other described models (37, 38). The model presented aims to demonstrate differences in sample behavior in a comparative aspect rather than in absolute duration of aneurysm growth. Nevertheless, even with such an interpretation, it should be assumed that the time of onset of proportional pathological changes in the scaffold structure will significantly differ for reinforced and unreinforced cases.

The concept of creating a reinforcing layer to improve the mechanical properties of tubular objects, including vascular scaffolds, has been realized in only a few studies. Authors tend to use helical forms as a component that improves the mechanics of the object under study. For instance, in Janke et al. (33), a resorbable monofilament suture material was formed into a double spiral to support a collagen scaffold (33). The study showed comprehensive research of the obtained devices—mechanical tests, fatigue, compression, degradation in an enzyme solution *in vitro*. Of course, it is impossible to directly compare the results of our study and the work of Janke et al. due to differences in designs and materials, but the use of a double helix as the most stable vascular prosthesis framework seems relevant. A similar study, led by Huo et al. (39), which subsequently became the basis for a more global mathematical study of support constructions (2018) (26), also attempts to create a frame of a double spiral using a similar principle—FDM-thermal extrusion of the polymer PCL. The authors evaluate the mechanical response of the helix and verify numerical models during a similar experiment with justification of the main dependencies on pitch, diameter, and filament properties. The authors come to similar conclusions: interconnected spiral structures demonstrate great potential as implants for tubular organs, especially in terms of mechanical properties: flexibility and stiffness.

The most successful practical implementation of reinforced vascular prostheses is described in Spadaccio C. et al. (20), who performed 10 interventions on rabbits (20). Developing the idea and technology of Centola M et al. (40), the researchers managed to implant samples into the descending aorta below the renal arteries as an “end-to-side” bypass. The implanted shunt functioned, preserving lower limb perfusion for four weeks. The hybrid vascular prosthesis had a PCL spiral reinforcement, mounted on a tubular PLLA polymer, manufactured using electrospinning technology.

Overall, our study and the cited literature demonstrate that bi-layered vessels, combining a scaffold and external reinforcement, show satisfactory *in vitro* experience for improving mechanical properties. This approach allows the use of a base with a low modulus of elasticity, which requires protection of its shape in the early postoperative stage until cell colonization by the recipient and the development of a sufficient amount of extracellular matrix. Depending on the objective, it is possible to create either a temporary (PCL) or permanent (PVDF) external support for the scaffold. In the case of PCL, a more detailed *in vitro* (and subsequently *in vivo*) investigations into the rate and degree of helix property degradation are essential. This would help determine the properties and geometry where the helix does not lose its reinforcing function before a required period. For PVDF, which provides support throughout the entire “life” of the scaffold, there will undoubtedly be questions about the durability of the helices under prolonged cyclic loads. For this kind of study, numerical and physical fatigue experiments are needed, taking into account the function of the structure over hundreds of millions of load cycles. Both of these research directions could be subjects of future work and bring the use of tissue-engineered vascular prostheses based on decellularized material closer to broader clinical practice.

## 5. Conclusion

Our study demonstrates the promising results of the FDM-thermal extrusion reinforcement of biological vascular scaffolds with polymer helices. The correct selection of polymers, combining properties of biocompatibility and thermoplastic extrusion capabilities, allows for improving the radial strength of decellularized scaffolds while maintaining original compliance and longitudinal rigidity under simulated physiological loads, including long-term ones. The presented method makes it possible to create a reinforcing layer from both biodegradable (PCL) and non-degradable (PVDF) polymer materials for vascular scaffolds.

## 6. Limitations

It is worth noting a significant limitation of the current study—the absence of an evaluation of the scaffold's bending stiffness. The main focus of the work was on researching the modification of only the radial stiffness of the hybrid construction; however, during its work, the device will also undergo some bending loads. Under such loading, there is a compression of the central lumen and potential disturbances in the integrity of the spiral structure, so such an evaluation should be included in the list of comprehensive justifications for the reinforcement approach in the future and serve as a basis for future work.

The second limitation of this study is the simplified biomechanical model of the vascular wall. Even though we introduce a component of plastic deformation and the associated degradation of the elastic modulus into it, this description does not account for other mechanisms of material property changes—the destructive action of immune cells or some restoration of properties due to the scaffold being populated by recipient cells. Moreover, the model does not employ the anisotropy of properties caused by the orientation of collagen and elastin fibers, which should affect the amplitudes of pathological bulging, however, potentially not alter the comparative picture—the reinforced scaffold should also effectively resist the development of aneurysmal bulges. More sophisticated models are available; the possibility of their application in this context should be considered further.

## Data Availability

The datasets presented in this study can be found in online repositories. The names of the repository/repositories and accession number(s) can be found below: (GrabCAD repository) https://grabcad.com/library/biological-scaffolds-1.
