# Supplementary material for: Enhancing decellularized vascular scaffolds with PVDF and PCL reinforcement: a fused deposition modeling approach
Source: Front Cardiovasc Med. 2023 Nov 29;10:1257812. doi: 10.3389/fcvm.2023.1257812 (PMC10716200; doi:10.3389/fcvm.2023.1257812)
Supplement: Supplementary file 1 [file Table1.docx]

**Supplementary materials 1**

Table 1. Compliance evaluation of scaffolds

|  | **PVDF** | **PCL** | **Control** | **p-value**  **PVDF vs Control** | **p-value**  **PCL vs Control** |
| --- | --- | --- | --- | --- | --- |
| **In vitro compliance, %** | | | | | |
| 50-90 mmHg | 2.2  [1.65-2.45] | 1.8  [1.5-2.3] | 1.7  [1.42-2.33] | 0.73 | 0.95 |
| 80-120 mmHg | 1.9  [1.7-2.2] | 1.7  [1.6-1.9] | 1.5  [1.35-1.75] | 0.36 | 0.5 |
| 110-150 mmHg | 1.6  [1.3-1.8] | 1.4  [1.2-1.7] | 1.3  [1.05-1.5] | 0.76 | 0.58 |
| **In silico compliance, %** | | | | | |
| 50-90 mmHg | 1.2  [0.9-1.6] | 1.8  [1.5-1.9] | 1.4  [1.2-1.7] | 0.6 | 0.46 |
| 80-120 mmHg | 1.7  [1.4-1.9] | 1.6  [1.5-1.8] | 1.1  [1-1.4] | 0.12 | 0.21 |
| 110-150 mmHg | 1.6  [1.4-1.9] | 1.6  [1.5-2.5] | 1.5  [1.3-1.8] | 0.75 | 0.53 |

Note: Data support the Figure 7.

Table 2. Investigation of mechanical properties for both directions (longitudinal and transverse)

|  | **PVDF** | **PCL** | **Control** | **p-value**  **PVDF vs Control** | **p-value**  **PCL vs Control** |
| --- | --- | --- | --- | --- | --- |
| **Ultimate tensille strength, MPa** | | | | | |
| 50-90 mmHg | 2.81  [2.66-3.45] | 2.27  [1.86-4.01] | 3.69  [3.41-3.73] | 0.4 | 0.53 |
| 80-120 mmHg | 3.14  [2.8-3.64] | 1.8  [1.64-2.12] | 1.07  [0.7-1.23] | **0.012** | **0.037** |
| **Max tension, %** | | | | | |
| 50-90 mmHg | 88.6  [84.2-90.4] | 90.2  [83-90.6] | 95.2  [90-96.6] | 0.29 | 0.75 |
| 80-120 mmHg | 126.11  [108.28-127.39] | 103.18  [99.36-108.28] | 121.02  [112.74-129.94] | 0.17 | **0.047** |

Note: Data support the Figure 7.
