# Supplementary figures and images for: Enhancing decellularized vascular scaffolds with PVDF and PCL reinforcement: a fused deposition modeling approach
Source: Front Cardiovasc Med. 2023 Nov 29;10:1257812. doi: 10.3389/fcvm.2023.1257812 (PMC10716200; doi:10.3389/fcvm.2023.1257812)

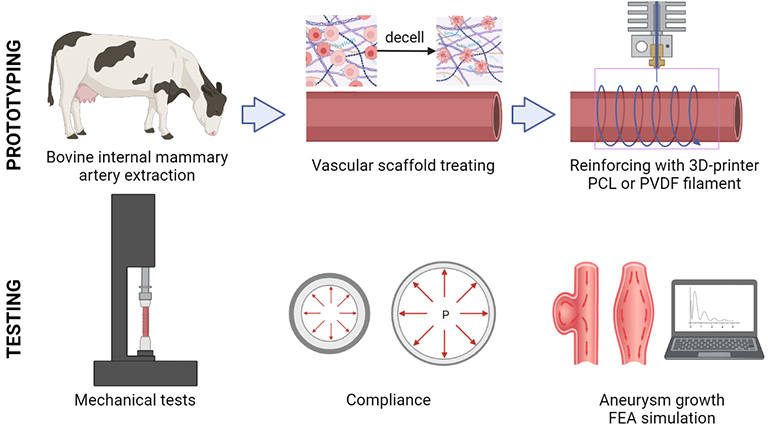

Supplement: Supplementary file 2 [file Image1.jpeg]
